# Supplementary material for: The E3 ubiquitin-protein ligase MDM2 is a novel interactor of the von Hippel–Lindau tumor suppressor
Source: Sci Rep. 2020 Sep 28;10:15850. doi: 10.1038/s41598-020-72683-3 (PMC7522254; doi:10.1038/s41598-020-72683-3)
Supplement: Supplementary file 1 — Supplementary information. [file 41598_2020_72683_MOESM1_ESM.pdf]

The E3 ubiquitin-protein ligase MDM2 is a novel interactor of the von Hippel-Lindau tumor suppressor

Antonella Falconieri[1#], Giovanni Minervini[1#], Raissa Bortolotto[1], Damiano Piovesan[1], Raffaele Lopreiato[1], Geppo Sartori[1], Maria Pennuto[1,2], Silvio C.E. Tosatto[1*]

[1] Department of Biomedical Sciences, University of Padova, Viale G. Colombo 3, 35121, Padova, Italy.

[2] Veneto Institute of Molecular Medicine (VIMM), 35129, Padova, Italy

[#] Contributed equally;

[*] Corresponding author.

Supplementary material

Supplementary Table 1

Plasmid Name	Primer Name	n°	sequence (5'-3')	Protein expressed
pGAD – pVHL30	VHL30 For	1	ggaggccagtgaattcATGCCCCGGAGGGCGGAGAA	Gal4AD-pVHL30 (1-213)
	VHL30Rev	2	cacccgggtggaattgTCAATCTCCCATCCGTTGAT	
pGAD – pVHL19	VHL19 For	3	ggaggccagtgaattcATGGAGGCCGGGCGGCCGCG	Gal4AD-pVHL19 (54-213)
	VHL30Rev	2		
pGAD – pVHL β	VHL19 For	3		Gal4AD-pVHLβ (54-157)
	VHL-β Rev	4	cacccgggtggaattgCTAAGTATACACTGGCAGTGTGATATTGGC	
pGAD – pVHL α	VHL-α For	5	ggaggccagtgaattcCTGAAAGAGCGATGCCTCC	Gal4AD-pVHLα (158-213)
	VHL-α Rev	6	cacccgggtggaattgTCAATCTCCCATCCGTTGATGTGC	
pGAD – pVHL-Nt	VHL30 For	1		Gal4AD-pVHL-Nt (1-53)
	VHL-Nt Rev	7	cacccgggtggaattgTCACTCCTCCTCGGCGCCCA	
pGAD – pVHL (1-157)	VHL30 For	1		Gal4AD-pVHL (1-157)
	VHL157Rev	8	cacccgggtggaattg TTAAGTATACACTGGCAGTGTGATATTGG	
pGBK – MDM2 (1-491)	MDM2 For	9	catggaggccgaattcATGGTGAGGAGCAGGCAAATGTGCAATACC	Gal4BD-MDM2 (1-491)
	MDM2 Rev	10	ggatccccgggaattgttaGGGGAAATAAGTTAGCACAATCATTTGAATTGG	
pGBK – MDM2 (1-150)	MDM2 For	9		Gal4BD-MDM2 (1-150)
	MDM2 (1-150) Rev	11	ggatccccgggaattgTTATGAAGATGAAGGTTTCTCTTCCTGAAGC	
pGBK – MDM2 (151-350)	MDM2 (151-350) For	12	catggaggccgaattcCATTTGGTTTCTAGACCATCTACCTC	Gal4BD-MDM2 (151-350)
	MDM2 (151-350) Rev	13	ggatccccgggaattgTTAGTTTTCCAGTTTGGCTTTCTCAGAGATTTCC	
pGBK– MDM2 (351-491)	MDM2 (351-491) For	14	catggaggccgaattcTCAACACAAGCTGAAGAGGGCTTTGATGTTCC	Gal4BD-MDM2 (351-491)
	MDM2 Rev	10		
pGBK –	MDM2 (434-491)	15	catggaggccgaattcGCCATTGAACCTTGTGTGATTTGT	Gal4BD-

MDM2 (434-491)	For		CAAGG	MDM2 (434-491)
	MDM2 Rev	10		
pGBK – MDM2 (351-433)	MDM2 (351-491) For	14		Gal4BD-MDM2 (351-433)
	MDM2 (351-433) Rev	15	ggatccccgggaattgTTAATTAAGGGGCAAACTAGATTCCACACTC	
pGBK – MDM2 (351-452)	MDM2 (351-491) For	14		Gal4BD-MDM2 (351-452)
	MDM2 (351-452) Rev	16	ggatccccgggaattgTTAATGGACAATGCAACCATTTTTAGGTCG	
pGBK – MDM2 (329-433)	MDM2 (329-433) For	17	catggaggccgaattcTGGGCCCTTCGTGAGAATTGGCTTCC	Gal4BD-MDM2 (329-433)
	MDM2 (351-433) Rev	15		
pGBK – MDM2 (329-452)	MDM2 (329-433) For	17		Gal4BD-MDM2 (329-452)
	MDM2 (351-452) Rev	16		
pCDNA3.1 flag-MDM2			Purchased by Genescript cat n: OHu28568	Flag-MDM2 (1-491)
pCDNA3.1 flag-VHL30			Purchased by Genescript cat n: OHu23297	Flag-VHL30 (1-213)
pCDNA3.1 HA-VHL30	HA-VHL For	18	taccgagctcggatcATGGAGTACCCATACGACGTACCAGATTACG	HA-VHL30 (1-213)
	HA-VHL Rev	19	gatatctgcagaattTCAATCTCCCATCCGTTGATGTGCAATGCGC	
pCDNA3.1 HA-VHL19	HA-VHL For	18		HA-VHL19 (54-213)
	HA-VHL Rev	19		

Table S1. Oligonucletides used in this study.

List of primers used for vector cloning and construction.

Supplementary Figure 1

A

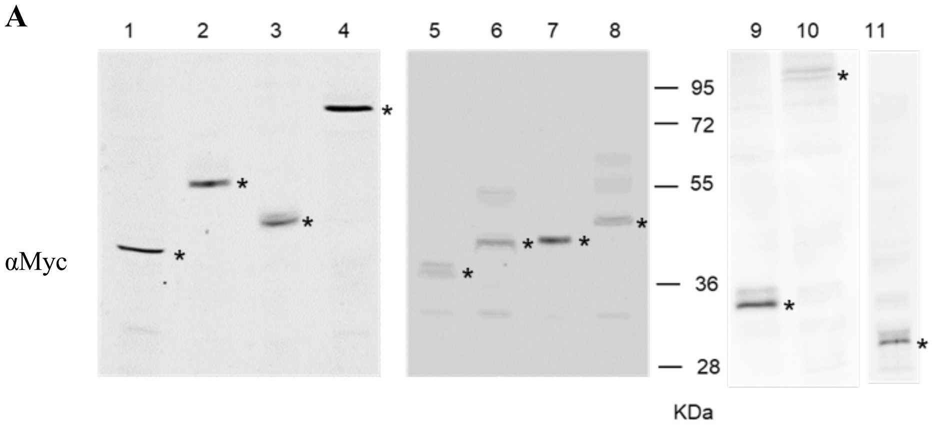

Legend:
1: MDM2 NT (aa 1-150)
2: MDM2 midi (aa 151-350)
3: MDM2 CT (aa 351-491)
4: MDM2 ΔCT (aa 1-350)
5: MDM2 (aa 351-433)
6: MDM2 (aa 351-451)
7: MDM2 (aa 329-433)
8: MDM2 (aa 329-451)
9: MDM2 (aa 434-491)
10: MDM2 full-length (aa 1-491)
11: empty (Gal4-BD, aa 1-147)

B

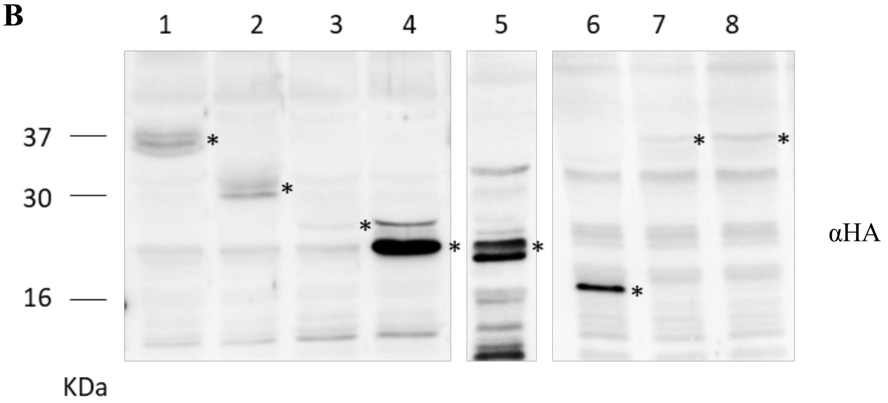

Legend:
1: VHL30 (aa 1-213)
2: VHL19 (aa 54-213)
3: VHL β (aa 54-157)
4: VHL Nt (aa 1-53)
5: VHL α (aa 158-213)
6: empty (Gal4-AD, aa 768-881)
7: VHL (aa 1-157)
8: VHL (aa 1-157)

Figure S1. Expression of Gal4-fusion proteins in yeast.

A) Western blotting (WB) analysis of total protein extracts of yeast strains expressing the Myc-tagged MDM2 full length and fragments fused to Gal4 DNA binding domain (Gal4-BD). Although MDM2 has a predicted MW of 55 kDa, in WB it runs around 90 kDa, as previously reported (Erhardt. P.,et al. 1997 JBC 272:15049-15052). Gal4-BD + linker have a calculated MW of 21 kDa and runs around 28 KDa. As expected, full length MDM2 run around 110 kDa. Similarly, all fragments in WB have a MW higher than MW calculated.
B) Western blotting (WB) analysis of total protein extracts of yeast strains expressing HA-tagged pVHL full length and fragments fused to Gal4 DNA activation domain (Gal4-AD).

Myc-tagged and HA-tagged proteins were detected with specific anti-Myc and anti-HA antibodies. Shown is one experiment representative of 3 (n=3). Asterisks indicate specific bands.

Supplementary Figure 2

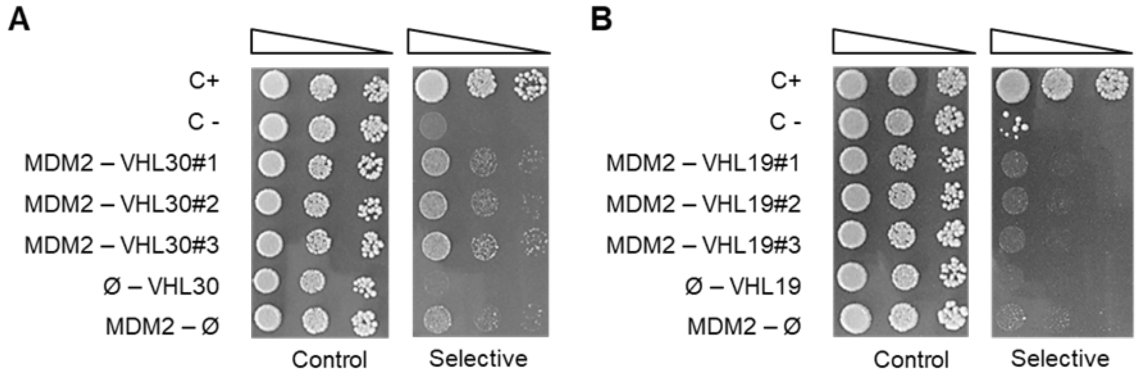

Figure S2. pVHL30 forms a complex with MDM2.

A-B) Y2H assays between full length pVHL30 and MDM2 (A), and full length pVHL19 and MDM2 (B) showed that pVHL30 specifically forms a complex with MDM2. (C+) positive control, (C) negative control, 3 clones co-expressing MDM2 (bait) and pVHL30 (prey). Expression of pVHL30 and MDM2 alone did not result in yeast growth in non-permissive medium.

Shown is one experiment representative of 3 (n=3).

Supplementary Figure 3

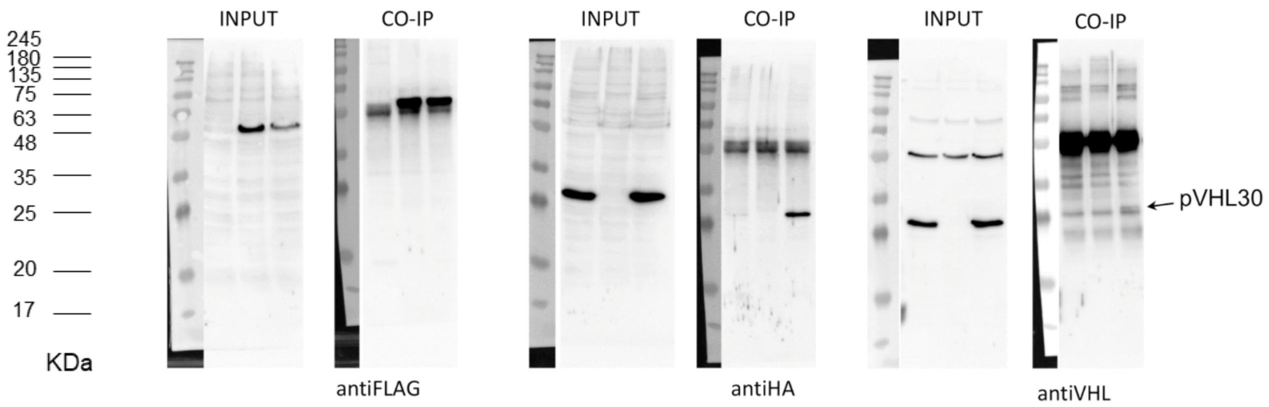

Figure S3. Uncropped full-length pictures of Western blotting membranes of Figure 2 panel B in the main text. Arrow indicates pVHL30 band recognized by anti-VHL antibody (n=3).

Supplementary Figure 4

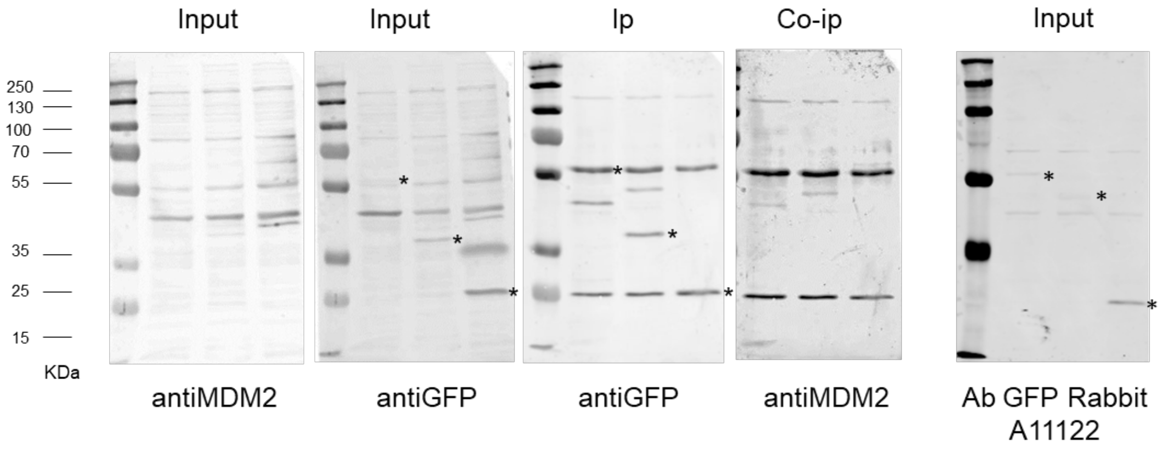

Figure S4. Uncropped full-length pictures of Western blotting membranes of Figure 2 panel C in the main text.

Uncropped full-length images of Western blotting membranes of figure 2 panel D in the main text. As additional control inputs were also analyzed using the anti-GFP antibody (A11122, Thermo Fisher Scientific). Asterisks indicate from left to right: pVHL30-GFP, pVHL19-GFP, GFP both in input and Ip panels (n=2).

Supplementary Figure 5

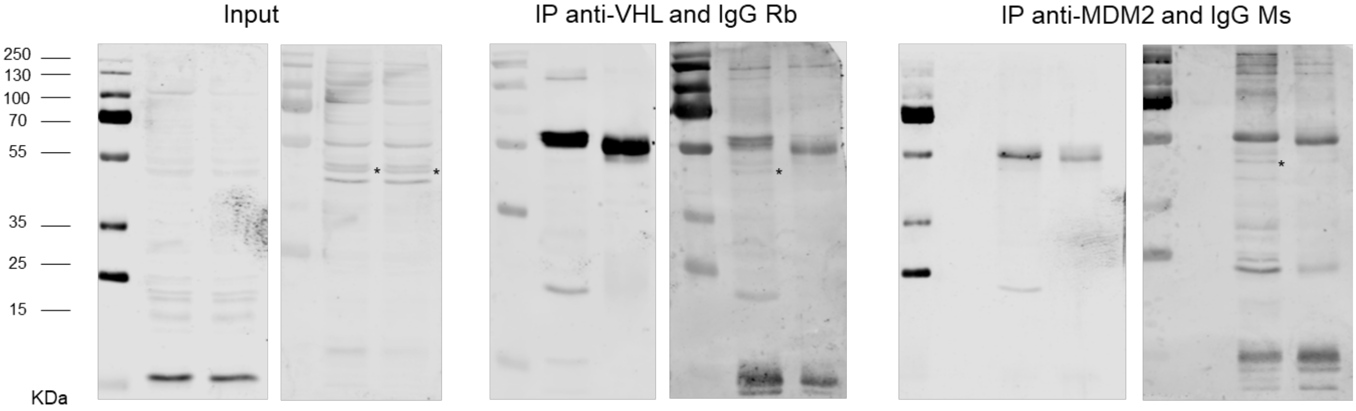

Figure S5. Uncropped full-length pictures of Western blotting membranes of Figure 2 panel D in the main text. Asterisks indicate bands corresponding to MDM2 protein (n=3).

Supplementary Figure 6

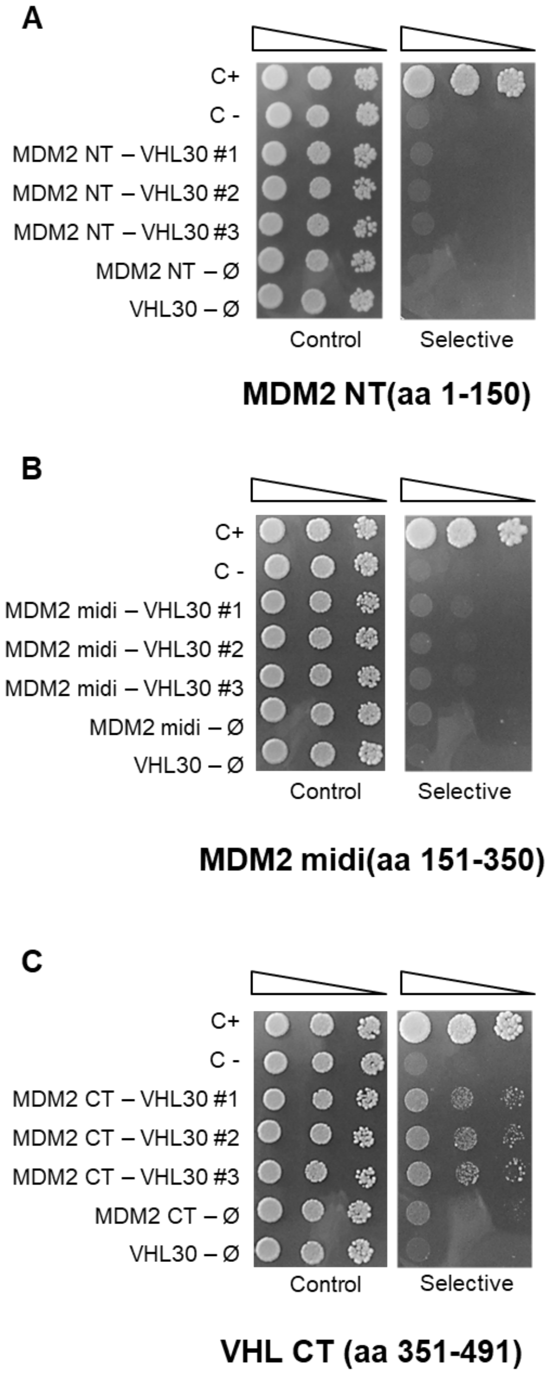

Figure S6. Uncropped images of figure 3 panel B in the main text.
A: MDM2 NT (aa 1-150); B: MDM2 middle (aa 151-350); C: MDM2 CT (aa 351-491). Shown is one experiment representative of 3 (n=3).

Supplementary Figure 7

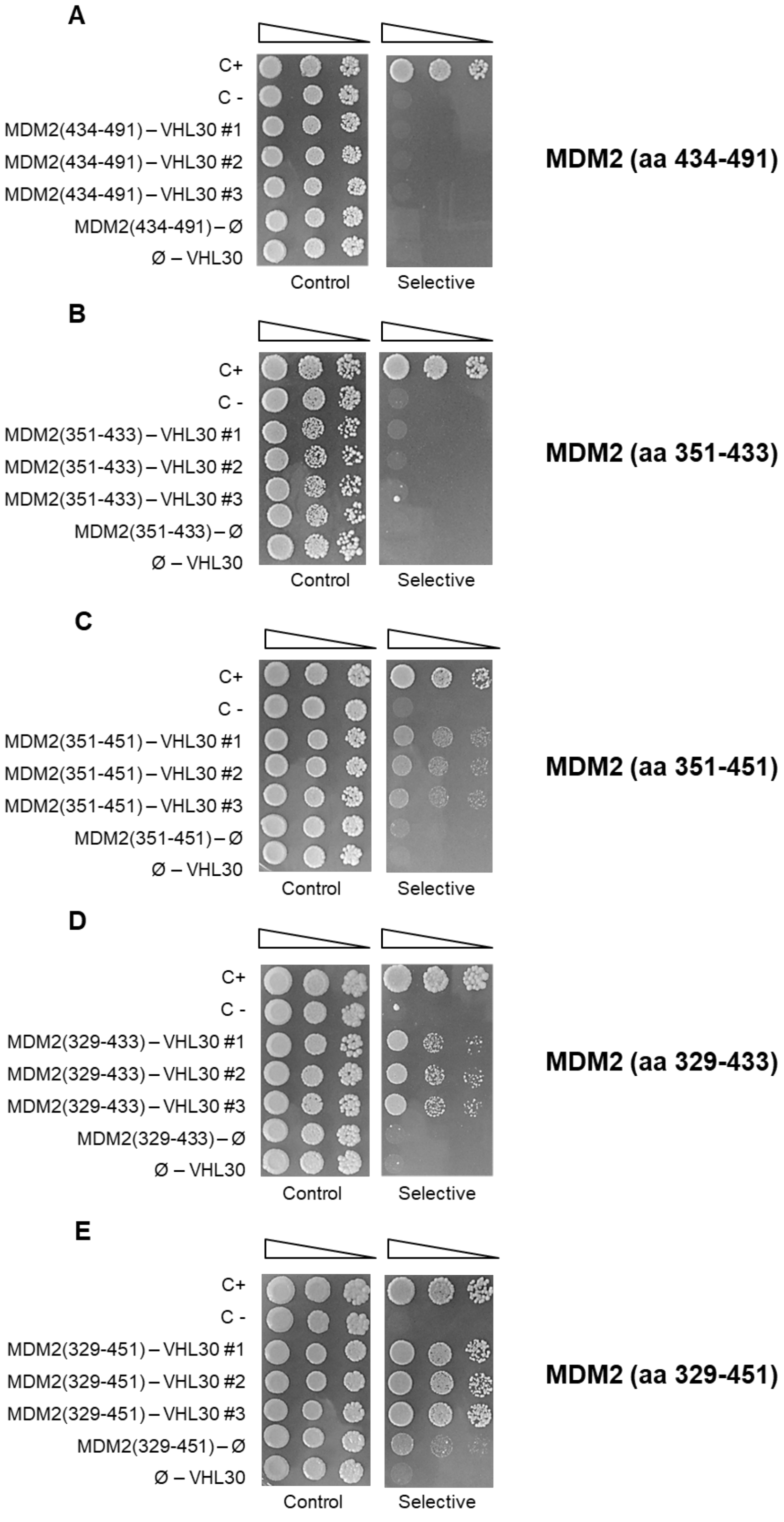

Figure S7. Uncropped images of figure 3 panel E in the main text.
A: MDM2 (aa 434-491); B: MDM2 (aa 351-433); C: MDM2 (aa 351-451); D: MDM2 (aa329-433) and E: MDM2 (aa 329-451). Shown is one experiment representative of 3 (n=3).

Supplementary Figure 8

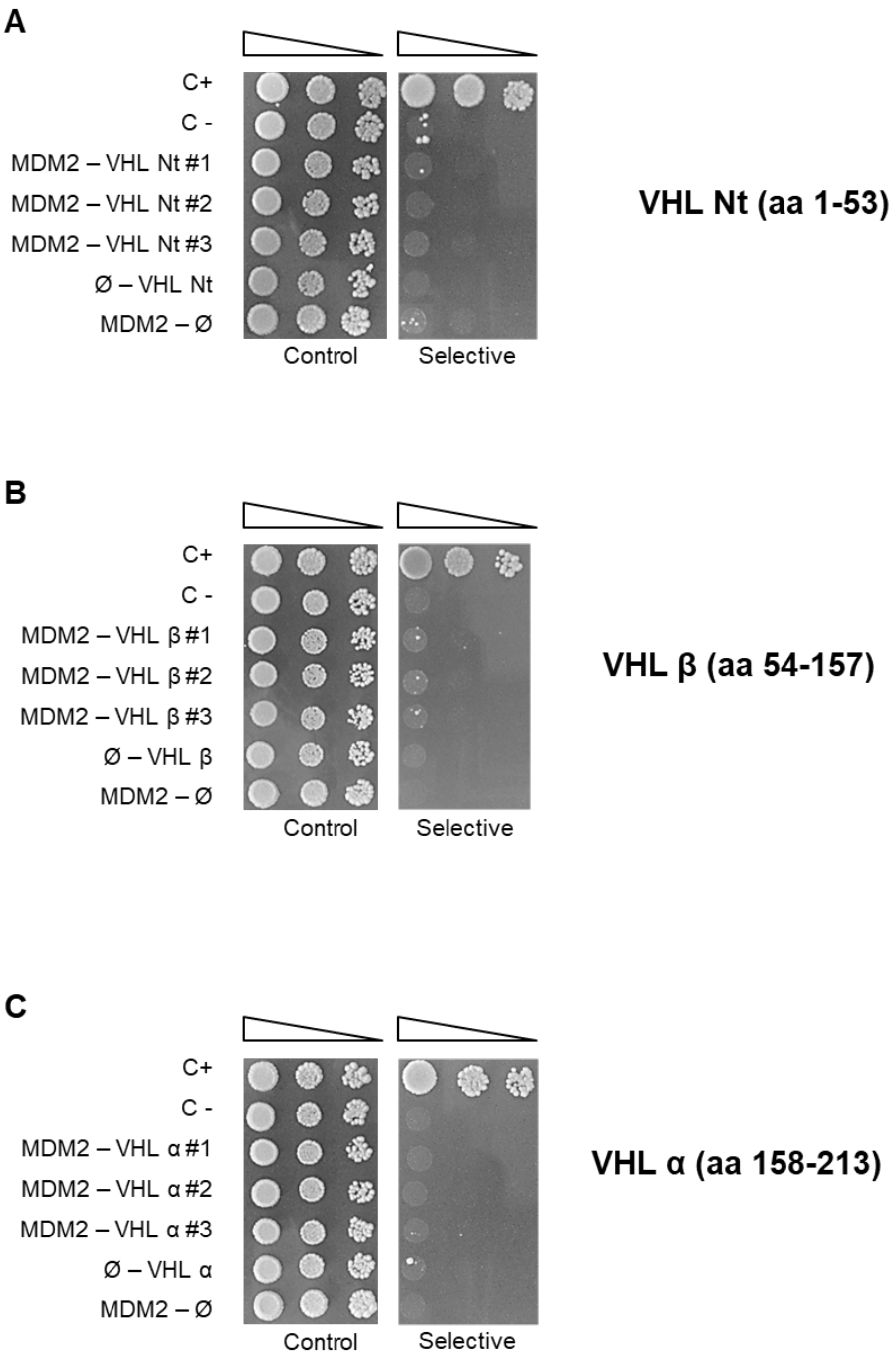

Figure S8. The N-terminus and β- and α-regions of pVHL30 do not associate with MDM2. A-C) Y2H assays analysis of interaction between full-length MDM2 and (A) pVHL Nt (1-53), (B) pVHL β domain (aa 54-157), and (C) pVHL α domain (aa 158-213). Shown is one experiment representative of 3 (n=3).

Supplementary Figure 9

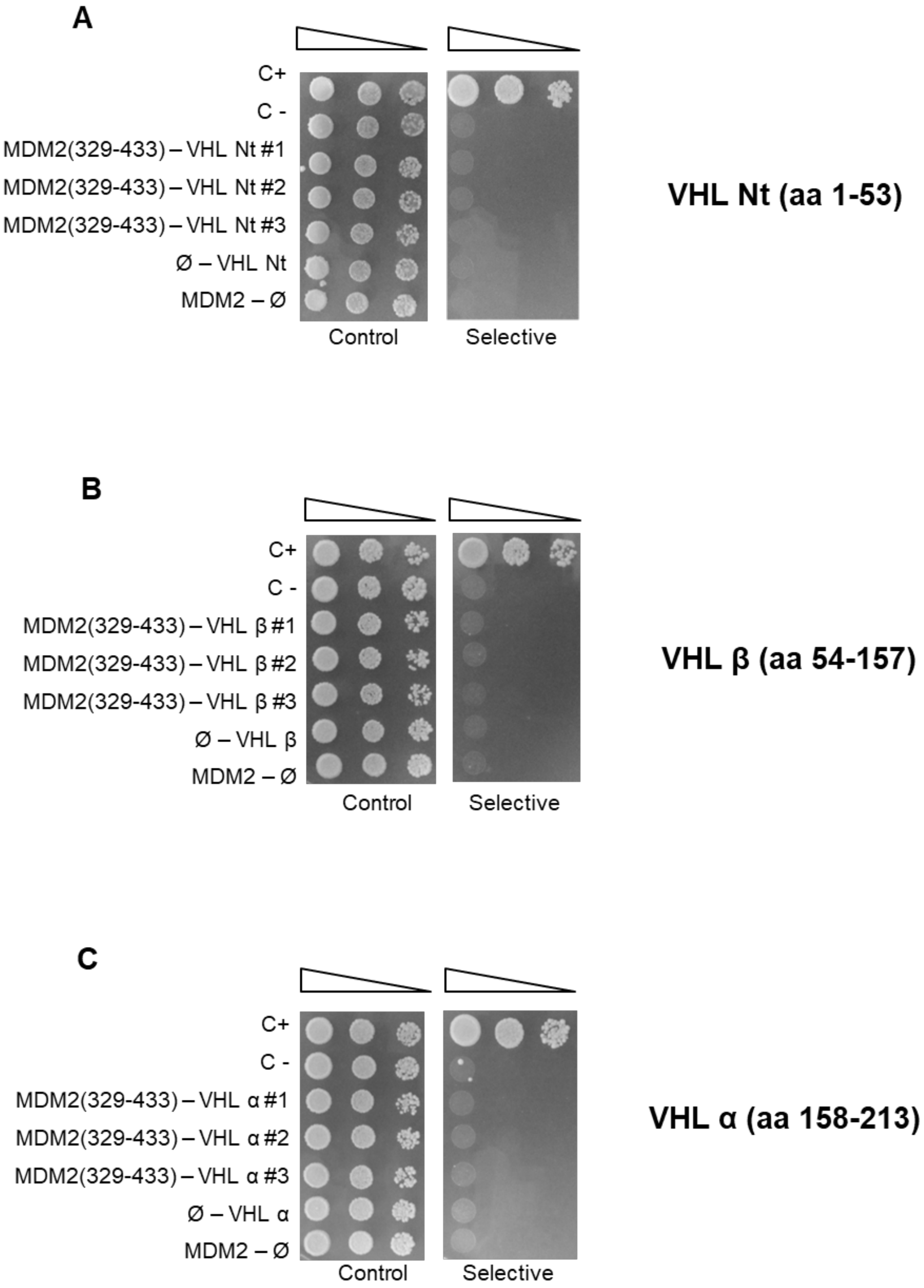

Figure S9. Uncropped images of figure 4 panel B in the main text.
A: VHL Nt (aa 1-53); B: VHL β domain (aa 54-157); C: VHL α domain (aa 158-213). Shown is one experiment representative of 3 (n=3).

Supplementary Figure 10

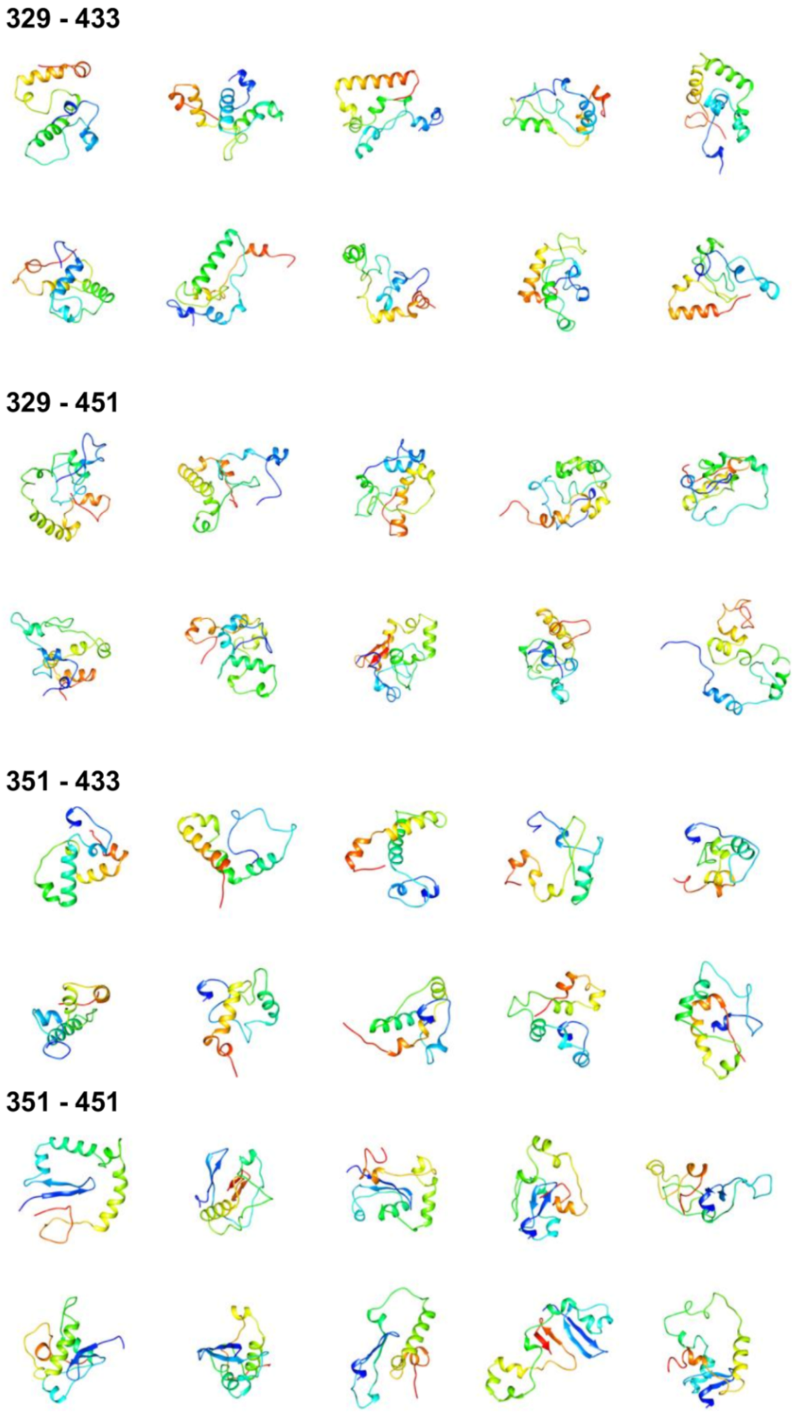

Figure S10. Overview of predicted 3D structure of each MDM2 fragment.

The best ten 3D structures calculated for the MDM2 fragments used during yeast two hybrid experiments. Structures are presented as cartoon and colored blue (N-terminal) to red (C-terminal).

Supplementary Figure 11

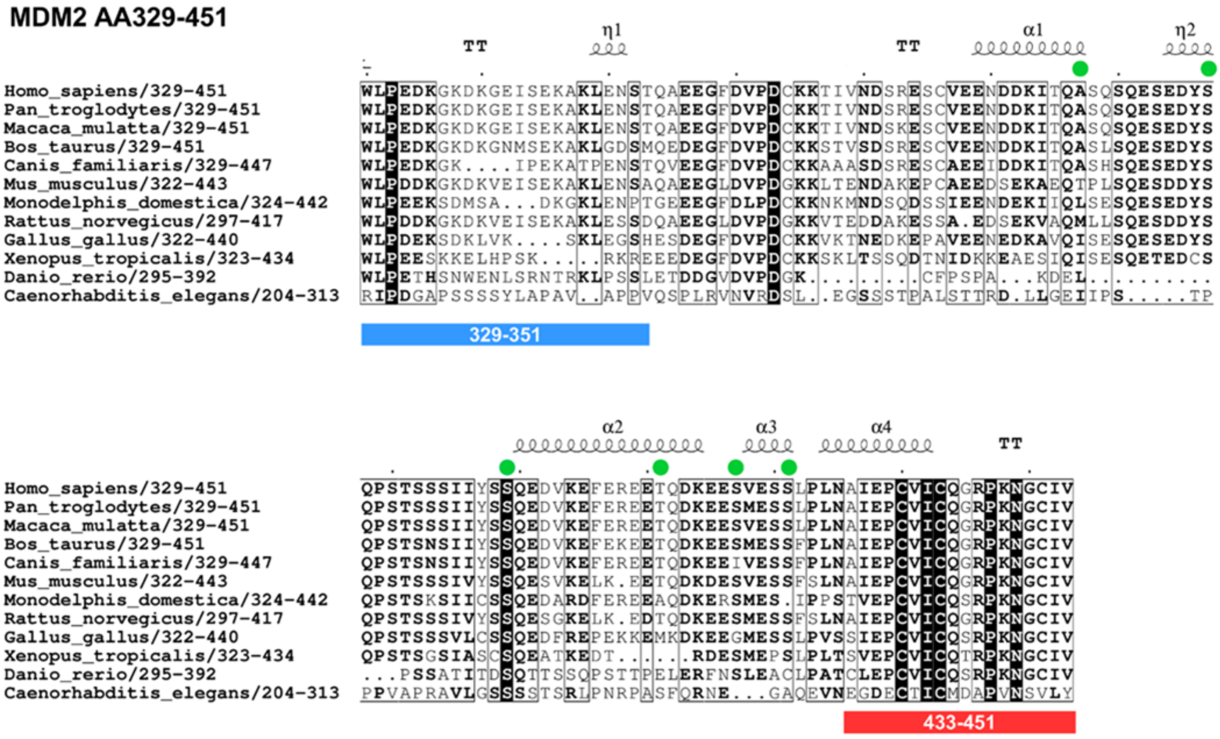

Figure S11. Multiple sequence alignment of MDM2 329-451 fragment.

Colored boxes represent the position of the two flanking regions presumed to drive MDM2/pVHL30 association. Green sphere highlight position of phosphorylation sites.

Supplementary Figure 12

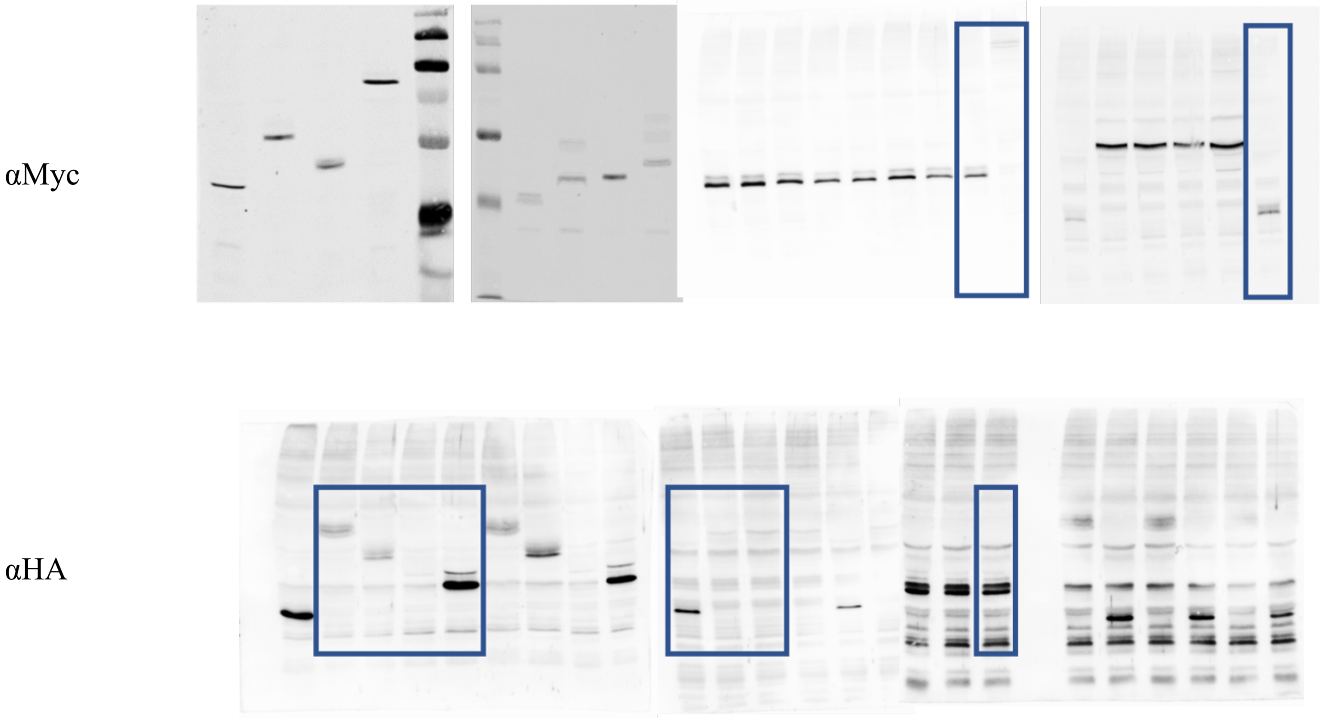

Figure S12 Western blot

Uncropped full-length pictures of western blot membranes referred to figure S1. Frames indicate the membrane area used in the cropped panel S1.(n=3)
